# Perspectives for Buck Kids in Dairy Goat Farming

**DOI:** 10.3389/fvets.2021.662102

**Published:** 2021-10-15

**Authors:** Ellen Meijer, Vivian C. Goerlich, René van den Brom, Mona F. Giersberg, Saskia S. Arndt, T. Bas Rodenburg

**Affiliations:** ^1^Department of Population Health Sciences, Faculty of Veterinary Medicine, Utrecht University, Utrecht, Netherlands; ^2^Royal GD, Department of Small Ruminant Health, Deventer, Netherlands

**Keywords:** buck kids, dairy goats, farm animal welfare, surplus offspring, farm animal ethics

## Abstract

To start milk production, dairy goats need to give birth at least once. While most female kids are reared to become the next generation of dairy goats, only a small proportion of male kids (buck kids) are reared with reproduction aims. The market for buck kid meat, especially within Northern European countries, is currently relatively small compared to the number of bucks born. Therefore, the purposes for buck kids are limited and a substantial proportion of buck kid meat is used for pet food. Due to the limited economic value of buck kids, farmers are faced with a dilemma. Although raising bucks costs more money than it yields, the birth of kids is a prerequisite for production of milk and should be seen as an investment for business-wise healthy dairy goat farming. In that perspective, dairy goat farmers have an ethical responsibility toward buck kids, as well. In this paper, we compare various scenarios of dealing with the issue of surplus male animals. We provide recommendations for the rearing of buck kids based on the sector‘s experience and current practice in the Netherlands. Reducing the number of surplus (male) offspring, e.g., by an optimized prolonged lactation management and/or by artificial insemination with sex-sorted semen, could alleviate the issue of low value buck kids. Killing surplus animals before or directly after birth, on the other hand, is met with increasing societal scrutiny. Initiatives to propagate a market for buck kid meat for human consumption are important to enable a suitable and sustainable production system. To maintain the health and welfare of goat kids, amongst other factors, sufficient and good quality colostrum, milk, and an appropriate diet as they grow older, needs to be provided. One option to assure the safeguarding of health and welfare of all goat kids are quality assurance schemes for milk production. These schemes make dairy farmers accountable for the health and welfare of all kids in the rearing period, including the provision of colostrum and adequate care for newborn buck kids. We conclude that the combination of reducing the number of surplus kids, increasing the demand for goat products, and quality assurance schemes that may help to safeguard the welfare of buck kids.

## Introduction

The dairy goat industry is an important sector in the Netherlands, which has grown substantially in the last decades. In 2010, ~98,000 female dairy goats were kept for milk production while this number rose to ~476,000 in 2020 (1). The majority of these goats are specialized breeds selected for milk production, mainly from the Saanen breed. To produce milk, a female goat needs to give birth at least once. The female offspring are mostly used as replacement for the dairy herd, thus replacing older and poorer performing goats. Most of the remaining female kids can be sold to other dairy farms within the Netherlands. The remaining surplus female kids are usually exported. In contrast, much fewer male kids (buck kids) are raised to serve reproduction, resulting in a surplus number of male offspring. Similar to other agricultural sectors specialized on female derived products (dairy, eggs), practical and ethical issues arise as there are only limited purposes for surplus male offspring (2–6).

Apart from the ethical concerns about the production of surplus male animals, potential welfare concerns rise, especially since the bucks have a limited economic value. Housing, health care, and slaughter cost a significant amount of money, and that investment is not returned in the revenue gained from the product yield from that individual animal. Therefore, buck kids may be at a higher risk for impaired welfare. Notably, these costs should be counted as total dairy goat farming management costs, and not per animal. Though limited scientific data exists for goats, the risks may be similar to those identified in male dairy calves (3, 4, 7). The British Veterinary Association and the Federation of Veterinarians of Europe have published recommendations and position papers regarding the production of surplus male animals, provide a framework for future research and practical solutions (2, 3).

Male offspring, e.g., male dairy calves, may be raised for slaughter (6). The market for goat meat in The Netherlands, however, is small (8), similar to that in other Northern and Western European countries. In Southern European countries, the market is relatively larger with a seasonal peak in goat meat consumption especially around Christmas and Easter (9). As the transport duration of young dairy goats to these countries is long and produces a risk of transmitting infectious diseases amongst the individuals, exporting these young dairy goats is undesirable (10–13). Therefore, virtually all surplus male goats stay in the Netherlands to be fattened for meat production, either for pet food or for human consumption. Most surplus buck kids nowadays are fattened on the farm they are born on. Specialized fattening facilities were commonplace within the Netherlands. However, currently the number of fattening facilities is rapidly declining, due to limited market opportunities and legal requirements (14). Currently, there are <5 fattening facilities remaining in the Netherlands (R van den Brom, 2021, personal communication), although they are still more common in other countries, such as Portugal, France and Italy (15).

Buck kids originating from breeds selected on milk yield and not on meat production, usually do not gain sufficient weight to be slaughtered for human consumption. As most buck kids are not used for breeding purposes, the rearing costs may exceed the benefits. Aside from structural and organizational costs (e.g., husbandry, marking and housing of the animals, health checks by veterinarians), the animals need sufficient nutrients to grow in the weeks before slaughter. When the costs of milk replacer were high, fattening facilities even charged the dairy farmer money to come and collect the kids (9). For many years, Dutch dairy goat farmers have been paying to get the buck kids to a fattening facility. A more recent trend is that buck kids are sold at a very young age for slaughter (for pet food) or are fattened on the farm of birth. Fattening the kids on the dairy goat farm where they are born circumvents the problem of transport and mixing animals from different farms, but may pose additional organizational and legal challenges to the farmer. Dairy goat farms may not have space to keep the bucks for fattening. Expanding the farm is not always possible, especially in densely populated countries such as the Netherlands, where local regulations may prohibit the expansion of existing farms. Housing conditions for kids raised on-farm may therefore be suboptimal, potentially resulting in reduced welfare. If the kidding season is stretched out over a longer period, kids from different ages may be housed together or in close proximity. This also increases the risk for transmission of infectious diseases.

In the case the buck kids are moved to a fattening facility, kids from a large number of farms are mixed into new groups and animals of different ages may be placed together. The mixing of kids from different farms and of animals of different ages increases the risk of social stress and the risk of transmission of infectious diseases. To provide buck kids a healthy start in early life, providing sufficient and good quality colostrum for passive immunity, umbilical disinfection immediately after birth, and proper hygiene should be practiced. Since the buck kids are not a significant source of income for the dairy goat farmer, and in many cases cost money, there may not always be sufficient attention for appropriate care, resulting in animals vulnerable to infection (16). Also, when there is a distinct kidding season during which workload is particularly high for farmers, there may not be enough time available to provide sufficient care for high-risk goat kids (VC Goerlich, 2021, personal communication).

The age at which the bucks are slaughtered can be divided into three groups. According to EU legislation, transportation from the farm to the slaughterhouse of goat kids of which the navel is not completely healed is prohibited. Additionally, kids under 7 days old must not be transported for more than one hundred kilometers (17). In practice, the youngest age at which kids are transported from the farm to the slaughterhouse therefore is between 7 and 14 days. According to the definition for the purpose of the carcasses of these animals, almost all of their meat is used for pet food, whereas no reliable statistics are available for the Netherlands. Kids that are raised for the Southern European market are fattened to a weight of around ten kilograms, which usually takes 3–4 weeks. After slaughter, the carcasses are exported. Finally, farmers may extend the fattening periods for 10 weeks to 5 months to produce “rosé meat” (similar to rosé veal meat from young calves which are not feed restricted to produce “white” veal). This meat is usually sold through short local chains such as artisan markets and is a niche product with an estimated 10% of the total goat meat market. The proportion of kids slaughtered at 7–14 days or 3–4 weeks is variable but roughly estimated equal around 45% (R van den Brom, 2021, personal communication). All in all, the major part of goat kids, including male kids, are either slaughtered at a very young age and used for pet food or slaughtered after ~1 month for human consumption.

There are approaches to alleviate the issues of surplus or low value male animals in general, and buck kids in particular. These approaches fall into three categories: (1) reducing the number of unwanted male offspring, (2) increasing the value of male offspring, and (3) safe-guarding the welfare of male offspring.

In this paper, the legal, ethical, animal welfare and practical implications of these approaches will be reviewed and discussed, with the current situation in the Netherlands as the starting point. The experiences with approaches to alleviate the problem of surplus goat bucks may be applied to other countries facing the same challenge.

## Evaluation Framework

### Legislation

All possible approaches to the problem must adhere to relevant legislation. EU law contains standards on welfare, husbandry, transport and killing of goats. These standards are described in EU Regulations and EU Directives. Regulations are binding legislative acts which are implemented in all member states. Directives describe goals which should be met by the individual EU members by implementing them in national law. Additional national legislation may apply in the different member states.

Minimum standards for the protection of animals kept for the production of food, wool, skin, fur or other farming purposes are laid down in Council Directive 98/58/EC (18). It states that owners or keepers of animals should ensure that their animals do not suffer any unnecessary pain or injury and that their species-specific physiological and behavioral needs are met. In light of possible methods to decrease the number of unwanted male offspring using assisted reproductive technologies such as artificial insemination, Paragraph 20 of the Annex in particular is relevant. It states that:

“*Natural or artificial breeding or breeding procedures which cause or are likely to cause suffering or injury to any of the animals concerned must not be practiced*.”

Council regulation No. 1099/2009 (19) establishes minimum rules for the protection of animals at the time of slaughter or killing. Chapter 2 Paragraph 3 states in general terms that:

“*Animals shall be spared any avoidable pain, distress or suffering during their killing and related operations. The methods referred to in Annex I which do not result in instantaneous death (hereinafter referred to as simple stunning) shall be followed as quickly as possible by a procedure ensuring death such as bleeding, pithing, electrocution or prolonged exposure to anoxia*.”

For the purpose of this article, Chapter 2 Article 4 point 1 in particular is relevant when considering acceptable methods for the killing of surplus animals. It states that:

“*Animals shall only be killed after stunning in accordance with the methods and specific requirements related to the application of those methods set out in Annex I. The loss of consciousness and sensibility shall be maintained until the death of the animal*.”

Additionally, according to Article 7 point 1:

“*Killing and related operations shall only be carried out by persons with the appropriate level of competence to do so without causing the animals any avoidable pain, distress or suffering.”*

Another factor that may influence the management of goat kids, is the legislation around the application of electronic ear tags described in Council Regulation No. 21/2004, Article 4 point 1 (20):

“*All animals on a holding born after 9 July 2005 shall be identified in accordance with paragraph 2 within a period to be determined by the Member State as from the birth of the animal and in any case before the animal leaves the holding on which it was born. That period shall not be longer than six months.”*

### Ethical Aspects

When applying moral standards to animal welfare issues, there are various ethical perspectives. The most common are the utilitarian perspective, the animal rights perspective, and the view with an emphasis on “ethics of care” (21, 22). The utilitarian perspective attempts to weigh the interest of all parties involved. In the case of surplus male goats, the interests of the farmer (such as time, money and effort involved) are weighed against the welfare needs of the buck kid. Seen from the animal rights perspective, animals have an intrinsic value of their own. If the concept of the intrinsic value is applied rigorously, it would be morally unacceptable to rear and kill animals for human consumption, and even more so producing “wastage.” From the ethics of care perspective, keeping animals, including keeping them for food production purposes, creates a relationship between these animals and the human keeper. From this relationship results a greater moral responsibility toward the animals in human care (22). It can thus be regarded as part of the human moral responsibility in general, and of producers of animal products in particular, to improve sustainability and reduce wastage of resources in animal production systems, as well as ensuring the welfare of the animals involved. In conventional animal production, the utilitarian perspective seems to be dominant. However, in recent years societal debate in Europe around animal production is increasing and the number of people adhering to the ethics of care or animal rights perspective seems to be increasing (23). In the future, the view on how animals should be treated may therefore be based on these perspectives.

### Animal Welfare

The aim of the welfare legislation is to ensure a minimum level of animal welfare. However, adherence to these rules does not necessarily mean optimal welfare is achieved. When judging the welfare implications of possible approaches to deal with surplus male goats, the first step is to clarify how welfare is approached. The Brambell committee (24) formulated the “five freedoms” as requirements for good welfare. Over the years, this definition has been modified by several researchers to provide space for adaptive capacity of animals and to place more emphasis on the animal experiencing positive emotions. For the purpose of this article, we use the concept proposed by Ohl and Van Der Staay (25):

*An individual is likely in a positive welfare state when it is able to adapt to the demands of the (prevailing) environmental circumstances, enabling it to reach a state that it perceives as positive*.

Buck kids may not be provided with the necessities to achieve a positive welfare state. Early weaning, insufficient intake of colostrum and nutrition, transport, and an unstable social environment pose threats to their ability to adapt to the challenges. Finally, methods of killing surplus animals on farm and at slaughter, should be evaluated and refined so that they impose as little suffering as possible.

### Practical Considerations

Apart from the legal, ethical, and welfare considerations outlined above, possible approaches to deal with surplus buck kids should also take practical considerations such as economics and sustainability into account.

Economic issues are, for example, that solutions that require a considerable time-investment from the farmer may require the recruitment of extra staff. Adapting housing to fatten bucks on farm can also require a considerable financial investment. For farmers, it is important that they are still able to obtain sufficient income. Some approaches may lower the margins on goat milk. Nevertheless, when producing goat milk, offspring is an unavoidable result of this production. The total management of a dairy goat farm consists of benefits (mainly milk, and sometimes some goats for trading purposes) and costs (housing, food, etc.). Buck kids can be seen as costs but are actually an investment to be able to produce milk. Therefore, farmers should be prepared to deal with the costs of raising these kids in a welfare-friendly and ethically acceptable way, just like they do with other production-associated costs.

“Sustainability” is a broad concept which can be defined as “acceptable now and in the future, related to consequences of functioning, morality of action and resource availability” (26). Producing animal products such as milk and meat put a strain on the environment. The ecological burden should be considered when judging potential approaches to deal with surplus male goats. The sustainability of raising buck kids for their meat needs to be checked on several attributes such as feed conversion efficiency and the use of resources such as water and land (27). High-quality feedstuffs (concentrates and roughage) and water are needed to sustain high-producing dairy goats, and to promote fast growth in meat-producing animals. If live animals or meat products are exported, the transport could increase the pressure on the environment (e.g., CO_2_ emissions or spread of infectious disease). Types of animal production systems where the majority of the products are exported to other countries are currently under debate in the Netherlands. This debate raises the valid question whether the Netherlands should, instead, focus its production on high-quality animal products, with high welfare standards, produced for the national or regional market. As goat meat is hardly consumed in the Netherlands, this type of production would not fit with that focus, unless the sector initiates actions to stimulate goat meat consumption in the Netherlands.

## Practical Solutions to the Male Surplus Dilemma in the Dairy Goat Industry

### Decreasing the Number of Male Offspring

#### Sexed Semen

Nowadays, it is possible to sort semen based on DNA content in many species (28–30). In goats, there is a substantial difference in DNA content between X and Y chromosomes in buck spermatozoa, allowing for a clear sorting of these two populations with an accuracy of around 90% (31). The challenge of producing kids with artificial insemination is 2-fold: the preservation of the sex sorted semen and the successful insemination of the female. Although kids have been produced using sex-sorted, cryopreserved semen, fertilization rates were low, even though the sexed semen was delivered by laparoscopic intrauterine artificial insemination. Out of eight female goats that were inseminated with X-enriched semen, only one kidded, while 4 out of 5 kidded with Y-enriched semen (32). While laparoscopic insemination may be improved heighten insemination success (33), the technique is prohibited in the Netherlands (34, 35). Generally, when laparoscopic insemination is used, the welfare of the receiving goat may be impaired due to stress and pain from the procedure.

The use of sexed semen always requires artificial insemination which, although sometimes used in some countries, is not being used on a large scale in dairy goat reproduction. Artificial insemination (regardless of whether sexed semen is used) offers some advantages, most importantly the possibility to introduce new genetic material onto a farm while maintaining a high level of biosecurity (36). On farms that use artificial insemination on their goats, synchronization and induction of estrus are performed before inseminating the goats. Synchronization of females, however, requires additional techniques such as hormone therapy, which is considered undesirable by many consumers as it is considered unnatural (37, 38). Also, the production of the hormone ‘pregnant mare serum gonadotrophin (PMSG)', which is used in most synchronization protocols for goats, involves sampling of live mares and is associated with substantial welfare issues for the horses involved (39).

From a practical point of view, sexing semen is, at the moment, still a costly and time-consuming practice. Sex sorting semen requires expensive flow-cytometers, while the processing speed greatly influences the accuracy of sorting. Moreover, conception rates with using sex-sorted semen are low which may necessitate multiple attempts before pregnancy is achieved (40). However, the financial consequences of using sexed semen may be partly offset by the reduction in costs for raising bucks, if technical results can be improved.

In conclusion, although the use of sexed semen is technically possible in goats and may reduce the number of surplus male offspring, similar as in dairy cattle (41). As artificial insemination with sexed semen will likely result in poor pregnancy rates, as sexed semen is only available in frozen form. The low conception rates and high costs preclude the routine use of this technique at this time, though methods may be improved (42). The use of hormones, however, associated with insemination with sexed semen could be problematic from a consumer point of view. Welfare issues for the mother goat may arise with laparoscopic insemination, which is not allowed in the Netherlands.

#### Genome Editing Techniques

In the future, genome editing techniques, in particular CRISPR/Cas9 systems, could provide additional methods to manipulate the sex of offspring. In pigs, knockout of the SRY gene using this technique has resulted in phenotypically female boars, although functionally these animals did not show heat (43). The authors suggested that targeting multiple genes on the Y chromosome during spermatogenesis to prevent the development of Y-chromosomal sperm could result in boars that only produce genetically and phenotypically female offspring. In goats, the CRISPR/Cas technique has successfully been used for genetic engineering (44), although not with the aim of producing only female offspring. Apart from the present technical challenges of using genome editing techniques (45), societal acceptance of these techniques may also pose a problem (46, 47).

#### Prolonged Lactation

In prolonged lactation [definition: lactation > 1 year without kidding, also referred to as extended lactation (48)], goats are not bred every year, and are sometimes bred only once during a lifetime. As a result of this, lactation continues for multiple years, leading to fewer offspring being born. As goats may give birth to one up to five kids at once, the reduction of the number of offspring depends on the duration of prolonged lactation.

Lactation curves within a prolonged lactation in goats are highly persistent, and goats may continue to be milked for 2–4 years (49) and even longer (R van den Brom, 2021, personal communication). When comparing milk yield of goats with an extended kidding interval of 24 months to goats with a 12-month kidding interval, the latter produced less milk from the 10th week of pregnancy (39th week of lactation) onwards, whereas extended lactation did not result in a decreased milk production (50). In the same study, milk composition was also analyzed. In late pregnancy (from week 12 onwards), the percentages of fat and protein were higher than in non-pregnant animals, while in the first 29 weeks of the second lactation they were lower than in the animals with prolonged lactation (50). The results of these studies thus suggest that prolonged lactation does not negatively affect milk yield.

Most health problems in older (multiparous) goats occur around kidding (51, 52). Pregnancy, parturition and the start of lactation are periods with a higher risk for health problems such as acetonaemia (twin lamb disease), hypocalcemia, endometritis, and mastitis. Multiparous, high-producing goats are particularly vulnerable to metabolic disease during the transitional periods around parturition (51, 52). The possibility of prolonged lactation in goats results in a limitation of the number of pregnancies during an animal's lifetime and may therefore improve health and welfare of the goat, alongside a reduced number of offspring produced.

On some farms, forced cessation of milk production (“drying off”) before the next parturition is a routine management practice. Although, often omitting the dry-off period is also practiced, but negatively influences colostrum quality (53), which in turn may negatively affect kid health. Although research on the effects of drying off on goat welfare is scarce, work on other ruminant species suggests that drying off may negatively impact welfare. Abrupt cessation of milking may result in pain (54) and a higher risk for intramammary infections (55). When feed is reduced to decrease milk production, hunger may cause additional welfare impairment. Therefore, reducing the number of times a goat has to be dried off may further benefit her welfare.

Prolonged lactation is mentioned as possibly associated with pseudopregnancy (48). With pseudopregnancy, aseptic fluid accumulates in the uterus (hydrometra) in the presence of a persistent corpus luteum. Pseudopregnancy results in anestrus and affected animals may show considerable abdominal distension, making it difficult to distinguish from true pregnancy. Pseudopregnancy can be diagnosed by abdominal sonography and treated by two administrations of prostaglandin F2α with 10 to 14 days in between (48). Although, to the best of our knowledge, it is has not been described that affected animals experience discomfort caused by pseudopregnancy, the necessity to treat animals with hormones costs money and may be perceived negatively by consumers.

In summary, prolonged lactation reduces the number of offspring and does not seem to negatively affect milk yield. It may have additional welfare benefits for the goats, since diseases associated with the transitional period are less frequent and potential discomfort from drying off, if applicable, is reduced. A potential trade-off may be that limited pregnancies may impair selection for breeding for animals with a highly persistent milk production, since there is less offspring from these animals. More research is needed on the (long-term) effects of extended lactation period concerning the health of the female and overall milk yield. Studies need to investigate whether this approach is a sustainable alternative to the current shorter lactation periods, considering its potential to substantially decrease the number of surplus offspring (56).

#### Humane Killing

Given the organizational effort to keep buck kids until transport to a fattening facility (with minimal revenue), humanely killing the kids shortly after birth may seem as an option. In the case this socially unwanted option is chosen, appropriate killing methods must ensure either immediate unconsciousness and loss of sensibility, or a pain and distress-free non-aversive induction of unconsciousness and insensibility. Furthermore, the duration of unconsciousness should be significantly longer than the total time required to ensure death of the animal (57). Apart from requirements that minimize suffering for the animals that are killed, additional requirements such as operator safety, economic viability and esthetical acceptability may be considered.

There are several methods available for the humane killing of goat kids on-farm (57, 58). Barbiturate overdose is generally viewed as a humane, safe and esthetically acceptable euthanasia method, but it requires a veterinarian to administer it. This makes it an economically less viable option for farmers. Additionally, the carcass has to be discarded at a fee to the rendering plant (59) and cannot be used for pet food.

Carbon dioxide depresses the reactivity of both respiratory and non-respiratory neurons, producing anesthesia and analgesia, and in higher concentrations it also causes hypoxia, leading to death (60, 61). Carbon dioxide challenge is used to provoke panic attacks in human research settings and may produce anxiety and pain before inducing anesthesia (62, 63). However, in goat kids undergoing reversible carbon dioxide anesthesia in concentrations up to 30%, it did not cause spontaneous aversion or conditioned place aversion (64). It may therefore be a suitable method to stun kids. Nevertheless, the limited data available and the need for specialized equipment preclude use on farm at this time.

Non-penetrating captive bolt devices cause concussion and cerebral damage that stuns, and depending on the extent of trauma, kills the animal. A study using a non-penetrating captive bolt device produced stunning in all of 200 neonatal goat kids included in the study, and after adjusting the positioning of the device after 42 stuns, all remaining 158 animals were both stunned and killed with one shot. These results indicate that non-penetrating captive bolt devices may be a suitable method for humane killing in neonatal goat kids, but that proper training and education of the operator is paramount when applying this technique (58). From the farmers point of view, there are some costs associated with the purchase of the device and the (blank) cartridges powering the device. Additionally, post-stun convulsions, although associated with the onset of an isoelectric EEG, may be esthetically unpleasant.

Regardless of a suitable humane killing method, there may still be ethical concerns regarding killing of unwanted goat kids *per se*. Even when the process of killing itself does not cause welfare issues, it may be argued that killing an animal prevents it from experiencing future welfare states (65). These welfare states may be either positive or negative. In the case of unwanted goat kids, one may argue that killing them humanely prevents them from experiencing future negative welfare. However, from an ethics of care point of view, the owner has a moral obligation to provide proper care for the animals and to prevent the occurrence of negative welfare states as much as possible. It can also be argued that animals have an intrinsic value that is violated by merely treating them as disposable by-products.

Consumer attitudes seem to be shifting in favor of production systems that do not kill surplus male animals, also in other farm animal species. As an example, in laying hen production, the male chicks were killed routinely after hatching using CO_2_ or maceration. This practice is subject to public concerns. Society prefers alternatives where the males are reared for slaughter (dual-purpose birds) or where eggs containing a male embryo are not incubated (47–49). Recently, Germany has decided to ban the killing of day-old male chicks from 2022 onwards (66) and other countries may follow. It may therefore also be of economic interest to look for alternatives for humane killing of unwanted animals.

### Increasing Value of Male Offspring

#### Increasing Demand for Goat Meat

Traditionally, goat meat is not in high demand in Northern Europe, even though it has several positive qualities. Compared to other red meats such as beef and lamb, it has a lower overall fat, saturated fat and cholesterol content, but more polyunsaturated fatty acids (67–70), making it a healthier option overall. Consumers unfamiliar with goat meat however are often unwilling to try it, because they expect it to be strong-flavored and inconvenient (71). As a consequence, goat meat represents only a small proportion of circa 1.5% of the yearly per capita meat consumption in the Netherlands (8).

There may be several ways to stimulate the consumption of goat meat in northern Europe. When asked about marketing strategies to increase consumption, consumers stated that they would like to see more information on the packaging label regarding health benefits, origin of the meat, production practices and traceability (71, 72). Also, the production of cured sausages based on male goat meat may be a good option, although this requires extra labor and may be too costly.

Increasing the availability and visibility of the product could help in the acceptance of goat meat (71, 72). In recent years, there has been promotion for meat from male “surplus” animals. By telling the story of these animals and by presenting attractive dishes based on products from male animals, more consumers may start to buy meat from male animals. From the laying hen sector, the Dutch Kipster system may inspire goat farmers in countries where buck kids have limited purpose. In this system, the male brothers of the laying hens are grown to 17 weeks of age and used for chicken burgers that are sold under a special label in the supermarket that also has exclusive rights to the Kipster eggs. Similar examples are currently developing in the Dutch goat sector. One of those examples is “Biogoatmeat” (73) in which Dutch organic farmers have united to promote the consumption of meat from surplus bucks from the organic goat milk industry through short, local chains. The meat is sold to restaurants and retailers, but also directly to consumers through a separate brand called “De Bokkenbunker”. Emphasis is placed on sustainability and animal welfare considerations. Biogoatmeat is also one of the participants in “Boktober” (and its international counterpart “Goatober”) (74, 75), an initiative to promote the consumption of goat meat during the month of October. Several interest groups are united within this initiative, which aims to familiarize consumers with goat meat by adding a goat dish to restaurant menus and by encouraging people to prepare goat meat at home.

#### Increasing Value of Goat Kids by Other Means

Traditionally, goats have not only been used to produce milk and meat, but also for their fiber and skins.

Cashmere is very fine wool with a diameter of <19 μm and is mainly used to produce high-quality clothing articles (76). In the past, some calculations have been made on the profitability of crossbreeding dairy goats with cashmere goats in an attempt to produce kids that produce cashmere fiber (77, 78), showing it to be a potentially profitable endeavor. However, the heritability of the desired low fiber diameter was negatively correlated with the heritability of the amount of cashmere wool that could be harvested from crossbred goats, complicating further selection on these traits (79). To our knowledge, no further attempts have been made to crossbreed dairy goats with cashmere goats to increase profitability of the offspring.

Leather from surplus buck kids is not being used on a large scale at the moment, although some small initiatives do exist (80).

### Safeguard Welfare of Buck Offspring

Regardless of the alternatives presented in previous paragraph, the challenge of surplus dairy goat buck kids is unlikely to be completely solved over a short period of time. Therefore, initiatives to safeguard the health and welfare of the kids that are produced are necessary.

There are several action points that could improve welfare of surplus bucks within existing production systems. These include ensuring the responsibility of the dairy goat farmer for the goat kids that are produced on the farm and improving the monitoring of mortality among goat kids.

To maintain the health and welfare of goat kids, amongst other factors, such as hygiene, housing, climate, sufficient and good quality colostrum, milk, and an appropriate diet as they grow older, needs to be provided, since they are essential. Colostrum quality and quantity are vital for a proper development of the immune system. Natural colostrum (either refrigerated or frozen) provides a much stronger boost to the immune system than commercially available artificial sheep colostrum. The peak in immune responsiveness of the goat kids was within 36 h for both natural colostrum sources and only after 30 days for the commercial colostrum (and the latter was 25 times lower) (81). Also, the following transitions from colostrum to milk or milk replacer and then to milk replacer and solid feed should be made with care. These transitions may impact health and resilience and may also be impacted by environmental conditions. A survey on the health and welfare status on 30 dairy goat farms in the USA, suggested that early kid management during birth to prevent illness/disease or mortality (e.g., warm and dry areas for kid rearing) was one of the main focus areas for future research (82).

An important prerequisite to monitor the well-being of goat kids was the ability to monitor mortality through improved identification and registration of young goats. Up until November 1st 2020, it was not mandatory for goat kids to be ear-tagged until they were 6 months old in the Netherlands. This hampered the ability to monitor mortality, as dead kids were just rendered to the animal carcass destructor in bulk. Under the new Dutch legislation, kids from dairy goat farms must be identified and registered within a 7 days after birth (83). At the same time, the sex of the kid must also be registered. Monitoring mortality provides the possibility to set limits for the maximal allowable mortality. Incrementally stricter limits are set to enforce stepwise reduction of mortality of goat kids.

As described previously, a common channel for Dutch dairy farmers for surplus buck kids was to have them collected by fattening facilities, usually at a cost for the dairy farmer. This meant there was not much incentive for the farmer to dedicate time and money to provide optimal care for these kids. In 2017, the Dutch Association of Dairy Goat Farmers (NGZO), together with the Netherlands Agricultural and Horticultural Association (LTO Nederland) created a plan of action to improve buck kid welfare (7). One of the starting points for this plan was the notion that the production of milk inevitably results in the production of offspring, and that the welfare of these animals is the responsibility of the dairy farmer up to the age of 21 days, regardless of whether the kids are fattened on farm or at a specialized fattening facility. Failure to ensure the welfare of the kids could ultimately result in withdrawal of the license to produce goat milk under the “Kwaligeit” label, the largest quality assurance scheme for goat milk in the Netherlands (84).

The number of fattening facilities in the Netherlands has declined rapidly. This may be partly due to the implementation of these stricter rules regarding the monitoring of kid welfare. There are, however, other factors that also influence the number of fattening facilities as well as the number of kids fattened on farm. An important factor in the Netherlands is legislation which prohibits the growth of goat farms. There are large local differences between municipalities on how strict this law is enforced; however, it is currently difficult both for fatteners and for dairy farmers to obtain permits to build facilities to keep dairy kids. There have been some changes in national legislation that have facilitated fattening on farm until 4 weeks of age, though restrictions on the expansion of farms continue to be an issue for farmers that want to fatten their own buck kids.

Another welfare-enhancing option, though critical for some health aspects, for both female and male kids would be to keep them in the dairy goat herd instead of moving kids to a fattening facility or separate them from their dams. Allowing goat kids access to their mothers for 24 h per day does not negatively affect somatic cell count in the milk or growth rate of the kids (85). Although the amount of marketable milk from the dams of the artificially reared group was somewhat higher, this was offset by the higher costs for labor and equipment in the artificially reared group. Notably, in intensive husbandry systems, the feasibility of keeping kids with their dams also depends on practical issues such as house design. From an animal welfare point of view, allowing goats and kids to perform their natural behavior (allowing them to adapt to the demands of the (prevailing) environment) and to develop the mother-offspring bond is favorable (86). However, weaning the kids from their mothers when their bond is already established and before the natural weaning age [which starts around 7 weeks (87)] may cause distress for both mother and kid, as indicated by e.g., increased call rates (88). An additional health risk in large groups may also be that newborn goat kids lose their mother in the group and start suckling from other females, thereby not taking up sufficient colostrum. Therefore, the benefits and risks for health and welfare of keeping goat kids in the dairy herd needs to be investigated in more depth.

## Discussion

The issue of surplus male animals in animal production is not limited to dairy goat farming. Similar challenges are present concerning the male offspring of dairy cows or laying hens. These challenges have their multi-faceted nature in common, as they entail ethical, animal welfare, practical, and economic aspects. Several stakeholders are involved such as farmers, dairy companies, retailers, consumers, and citizens. Therefore, holistic programs aimed at sustainable and animal welfare friendly strategies need to be developed, taking into account the various aspects of the issue and the needs of the various stakeholders. In this review we have described some fundamental issues concerning surplus buck kids, and introduced potential solutions.

To induce change, legislation plays an important role. In some countries (e.g., France and Germany), as an example, the ban of killing day-old chicks has put pressure on the sector to develop alternatives, such as sex determination of embryos and the destruction of eggs prior to birth. Nonetheless, the ethical question remains—whether it is acceptable to prevent male animals from being born or to kill them humanely shortly after birth. The dairy goat sector, as well as veterinarians, do not approve of routine humane killing of surplus male animals (2, 3, 7).

Overlooking the techniques to reduce the number of male offspring that is produced, optimization of prolonged lactation as management tool seems a very promising strategy with many positive effects and with a potential to reduce the number of offspring born by 50–75%. Other techniques, such as sex-sorting sperm, may also be a promising strategy, however, the technique is in need of further research and refinement. Although we described humane methods for killing surplus goat kids, this is the least desirable option as it poses ethical problems and is under increasing scrutiny by consumers and society, the dairy goat sector and veterinarians.

To increase demand for goat meat, marketing strategies need to be formulated to attempt to raise the attractiveness of male goats to the consumer and the profitability for the farmer. Examples of such marketing strategies come from the poultry industry and are slowly developing in the goat sector, as well. Although Southern Europe provide a market for 3–4-week-old goat kids from the Netherlands, dependence on this market leaves farmers vulnerable in case of contingencies, such as closed borders due to notifiable diseases. Therefore, the development of new, preferably local, markets is of importance to ensure the continued sale of goat meat.

The development of quality control programs that ensure appropriate care, have great potential to safeguard the welfare of buck kids. The Dutch example, where the dairy goat farmers made responsible for quality control, especially in combination with improved registration of young animals at the individual level shows great promise. Although economic aspects should be considered when deciding on suitable solutions, it is necessary that dairy farmers understand that when they want to produce milk and sufficient replacement animals, the production of surplus male offspring is unavoidable. The costs of appropriate care for these animals are just as much part of production costs as, for example, the cost of food for the adult goats. As an example, a Dutch farmer reported that in his case, the costs of fattening bucks were identical to the benefits, when growing buck kids to 8 kg (4 kg carcass yield): this results in an income of 14 Euro. The cost for 7 kg of milk replacer is also ~14 Euro (89).

Implementation of benchmarks such as maximum mortality within quality assurance programs for milk production help instill this notion. To help farmers reach these benchmarks, training and exchange of experience between farmers, with the aim to reduce kid mortality, would be helpful.

Finding a sustainable approach calls for the involvement of several scientific disciplines. Social scientists and economics need to advise on the economic aspects, animal welfare scientists, biologists, veterinarians and ethicists need to be involved in research on management of dairy goat herds, and techniques such as artificial insemination, optimalisation of prolonged lactation management, appropriate kid raising, and humane (on farm) killing. Farmers need to be supported and informed on their options.

In this paper, we discussed three main approaches to address the issue of unwanted male offspring in dairy goats: (1) reducing the number of unwanted male offspring, (2) increasing the value of male offspring, and (3) safe-guarding the welfare of male offspring. We evaluated effects on legal, ethical, welfare and practical aspects (Table 1).

**Table 1:** Expected effects of approaches to alleviate the problem of low value buck kids in the Dutch dairy goat sector.

	**Legal**	**Ethical**	**Welfare**	**Practical**
Decreasing the number of male offspring	0	+	0	+
Increasing the value of male offspring	0	+	+	?
Safe-guarding the welfare of male offspring	+	+	+	+

*+, positive; 0, neutral; ?, unknown*.

We conclude that all three approaches have positive effects on these aspects or that they are neutral. For legal aspects, the strongest effect is expected from safe-guarding welfare of male offspring, as this is a legal requirement. All three approaches are expected to have positive effects on ethical aspects, as a system where fewer male goat kids are produced and which focuses on increased value and product quality while paying attention for safe-guarding buck welfare is expected to be ethically more acceptable than a system where these aspects are taken into account to a lesser extent. Similarly, regarding welfare, positive effects are expected in a production system more focused on increases value and product quality and with attention for safe-guarding buck welfare. Regarding practical considerations, decreasing the number of male offspring (through an extended lactation period or the optimized use of artificial insemination) and safe-guarding buck welfare (through a quality control system) seem to be the most feasible. Approaches to increase the value and the market for buck meat in Northwestern Europe so far have had limited success, and could benefit from increased support of the retail sector.

## Author Contributions

TR conceived the original idea for the manuscript. EM drafted the manuscript. VG, RB, and TR revised the manuscript. RB, MG, SA, and TR provided critical remarks to improve the manuscript. All authors read and approved the final manuscript.

## Conflict of Interest

The authors declare that the research was conducted in the absence of any commercial or financial relationships that could be construed as a potential conflict of interest.

## Publisher's Note

All claims expressed in this article are solely those of the authors and do not necessarily represent those of their affiliated organizations, or those of the publisher, the editors and the reviewers. Any product that may be evaluated in this article, or claim that may be made by its manufacturer, is not guaranteed or endorsed by the publisher.
